# Different predation capacities and mechanisms of *Harmonia axyridis* (Coleoptera: Coccinellidae) on two morphotypes of pear psylla *Cacopsylla chinensis* (Hemiptera: Psyllidae)

**DOI:** 10.1371/journal.pone.0215834

**Published:** 2019-04-23

**Authors:** Yang Ge, Liu Zhang, Zifang Qin, Yang Wang, Pingping Liu, Shuqian Tan, Zhen Fu, Olivia M. Smith, Wangpeng Shi

**Affiliations:** 1 Department of Entomology, College of Plant Protection, China Agricultural University, Beijing, China; 2 Department of Entomology, Washington State University, Pullman, Washington, United States of America; Chinese Academy of Agricultural Sciences Institute of Plant Protection, CHINA

## Abstract

Pear psylla, *Cacopsylla chinensis* (Yang & Li) are present as two seasonal morphotypes in pear orchards where they, suck phloem sap, defoliate pear trees, and cause fruit russet. Despite the importance of natural enemies in psyllid control, the interactions between predators and the two seasonal morphotypes of psyllids remain poorly documented. Here we determined the predation efficiencies of the Asian lady beetle, *Harmonia axyridis* (Pallas) on the two psyllid morphotypes. Predation of *H*. *axyridis* on both morphotypes conformed to a Type II functional response: the proportion of consumed psyllids decreased with increasing prey densities. Predation efficiency of *H*. *axyridis* against the winterforms increased with temperature when measured from 8 to 25°C. Predation rate on the summerforms was significantly higher than that of the winterforms. This was linked to smaller body size, higher soluble protein level, thinner cuticle, and lower chitin content of summerform psyllids compared to winterform psyllids. Predation capacities of *H*. *axyridis* on both morphotypes indicated its potential as a biocontrol agent in psyllids management. Predation efficiency was higher on summerforms, likely due to the difference in body size, exoskeleton fragility, and nutritional value between the two morphotypes. Due to the Type II functional response of *H*. *axyridis* to both morphotypes of pear psylla, application of *H*. *axyridis* in pear orchards under suitable temperatures could be taken into consideration for suppression of *C*. *chinensis*, although further experiments conducted in field conditions are needed to validate our findings.

## Introduction

Pear trees are attacked by multiple herbivorous insects [1], including the phloem-sucking pear psyllid, *Cacopsylla chinensis* (Yang *&* Li). *C*. *chinensis* inflict devastating damage to trees and fruits in a number of East Asian countries, including China and Japan [2–3]. Damage caused by *C*. *chinensis* includes stunting and wilting of pear trees due to toxic saliva injecting into the trees [4]. Sooty mold fungus that lives on honeydew excreted by pear psylla reduces the photosynthesis rate of pear leaves [5]. Additionally, *C*. *chinensis* induces infection by plant pathogens [6–7]. Recently, it was reported that *C*. *chinensis* was responsible for vectoring phytoplasma, causing pear decline disease in Taiwan of China (PDTW) [6]. As one of the most impactful insect pests in the pear industry in East Asia, it causes great economic losses to growers [8]. However, current management of pear psylla in many countries remains primarily dependent on heavy usage of synthetic chemical insecticides [7, 9]. Meanwhile, pear psyllids have developed resistance to synthetic chemical insecticides likely due to their host specificity and high reproductive capacity [10]. From the socio-environmental perspective, application of chemical insecticides often raise concerns about health risks due to pesticide residues on crops and pesticide runoff into watersheds and other surrounding ecosystems. Therefore, eco-friendly alternative management strategies such as the incorporation of natural plant compounds [11], processed kaolin [12], and the introduction of natural enemies have attracted the attention of researchers and growers [8,13].

Application of predators to suppress psyllid populations in pear orchards has been documented in a number of studies [14,15]. Most of the previous studies has focused on the interactions of predators with summerform psyllids, while studies of biological control of winterform psyllids are less well documented. Like other *Cacopsylla* psyllids, *C*. *chinensis* exhibits seasonal dimorphism: the summerform is lighter in body color and smaller in body size than that of winterform [16–18]. In most areas of China, winterform psyllids are usually present from September to April, whereas summerforms are present during the rest of the year [19–20]. The population density of summerforms is highly dependent on the outbreak of overwintering populations in early spring [20]. Therefore, the early-season control of winterform psyllids should be considered as important as the control of summerforms in the year-round control of psyllid populations. Spiders, ladybeetles, mirid bugs, lacewings, syrphid larvae, and earwig larvae are thought to be effective predators of pear psylla in early spring [21–22]. To our knowledge, few studies have addressed early-season control of *C*. *chinensis* by natural enemies.

From the evolutionary aspect, a broad range of organisms, including insects, birds, and mammals, display morphological polymorphism as a result of adaption to predator-prey relationships [23–25]. To date, studies examining seasonal dimorphism of pear psylla have primarily focused on phenotypic plasticity and transformational conditions between summerform and winterform [26–28]. It remains unclear whether seasonal dimorphism of psyllids has an influence on predator-prey interactions or if it impacts the efficiency of biological control agents such as predators. In additional to morphological traits, biochemical characteristics could contribute to variation between the seasonal morphotypes that are adapted to different environments. Previous study has shown that the winterform pistachio psylla synthesize trehalose, a disaccharide molecule that acts as cryoprotectant to facilitate winter survival of the psyllids [29]. The winterform of pear psylla might exhibit physiological and biochemical characteristics which vary from the summerform. Many biochemical traits of prey can influence the preference and foraging behavior of predators [30–32], e.g. nutritional value and cuticle hardness. Studies have shown that predators select prey based on nutrients to meet their nutritional needs for survival, development, and reproduction [33–35]. Cuticle thickness or “hardness” of prey has also been found to influence foraging behaviors of invertebrate predators and many insectivorous vertebrate species [36–37].

Based on our previous field observation, *H*. *axyridis* is one of the most abundant species of predators of *C*. *chinensis* in pear orchards in Beijing (unpublished data). Therefore, we tested Asian ladybeetle, *Harmonia axyridis* (Pallas), a well-studied predator that is native to Asia [38]. Furthermore, *H*. *axyridis* has successfully colonized many countries where it was introduced as a biological control agent [38–39]. Large populations of *H*. *axyridis* have been found in fruit orchards outside of their native range, coinciding with the emergence of psyllids worldwide, e.g. pear orchards in Turkey [40] and citrus farms in Florida USA [41]. Despite the prevalence of *H*. *axyridis*, its control efficiency of *C*. *chinensis* has not been documented. Our objectives were to improve our understanding of the control potential of *H*. *axyridis* on two morphotypes of *C*. *chinensis* and to examine how the seasonal dimorphism of *C*. *chinensis* influences the predation capacity of *H*. *axyridis*. We achieved this by: 1) measuring the predatory capacity of *H*. *axyridis* on winterform *C*. *chinensis* at different temperatures in early spring (8–15°C), 2) measuring the differences of predatory capabilities of *H*. *axyridis* on two morphotypes of *C*. *chinensis* at 25°C and 3) assessing differences in physiological and biochemical traits of the two morphotypes of *C*. *chinensis* which relate to foraging behavior of *H*. *axyridis*.

## Materials and methods

### Insects

*Harmonia axyridis* used in the experiment were originally collected from bark cracks of cherry trees and stone crevices in Beijing Botanical Garden, Beijing, China (39°59'58"N 116°12'35"E). No specific permissions are needed to collect from this public park. *H*. *axyridis* is not on the list of endangered and protected species of China, is a very prevalent and abundant species, and we collected a very small quantity to avoid interruption of the local community. Upon arrival to the insectary, *H*. *axyridis* were kept in meshed cages (50 cm×50 cm×50 cm, 120 threads per 6.45 cm^2^ mesh), and fed with *Megoura japonica* (Matsumura) as a food source. *M*. *japonica* were mass reared on broad bean plants (*Vicia faba* L.) grown in plastic pots filled with a mix of nutrient soil and vermiculite (1:1). *H*. *axyridis* and *M*. *japonica* colonies were kept in an insectary at 25 ± 2 °C, 70 ± 5% relative humidity, and 16L: 8D photoperiod. The *C*. *chinensis* colony used in experiments was initiated from the field-collected individuals from a pear orchard in northwest of Beijing, China (40°08'14"N, 116°06'48"E). *C*. *chinensis* were then kept in enclosures which were comprised of pear trees (*Pyrus bretschneider*i Rehder) (>20 years old) covered by mesh (60 thread per 6.45 cm^2^ mesh). Pests and natural enemies were carefully removed from trees by beating the branches. No pesticides were used within the meshed enclosures. Live *C*. *chinensis* adults used in the experiments were collected from these enclosures by using an aspirator. Insects were taken back to the laboratory where they were reared on pear branches. The dates when we collected the two morphotypes coincided with peak population densities of psyllids in northern China [20, 42]. Psyllids were randomly selected from the established colonies and included both sexes. Psyllids were not sexed individually because of the large amount of psyllids used in the predation experiments. All insects used in the experiments were collected 48h before the beginning of the experiment and kept at the same temperature as their experimental conditions. *H*. *axyridis* were fed with the corresponding morphotype of psyllids for 24h to increase prey acceptance.

### Experimental design

#### Predation capacity of *H*. *axyridis* on two morphotypes of *C*. *chinensis*

Winterform adult psyllids were collected in March 2015 and summerform adult psyllids were collected in June 2015 (summerform individuals). Functional responses of *H*. *axyridis* were conducted to determine their predation capacity on winterform *C*. *chinensis* based on the methods described by Emami *et al*. 2014 and Pekár *et al*. 2015 [8,21] in a petri dish arena (9 cm diameter). Predators were starved individually for 24 hours in petri dishes as suggested by Nakamura 1977 [43]. Winterform adult psyllids were placed in the petri dish arenas first so that psyllids could disperse, ensuring that predators needed to search for their prey. A predator-free control was used to assess natural mortality of the psyllids. Six psyllid densities (10, 20, 40, 80,120,160 psyllids per petri dish) were used to measure the predator’s functional response at two temperatures (8 °C and 15 °C), with a relative humidity of 65 ± 5% and a photoperiod of 16L: 8D. The two temperatures were chosen to represent a typical early-season condition and a warmer condition [19,42]. Additionally, 8–15°C is also the average range of temperatures in which winterform psyllids lay eggs [19]. After the accurate densities of psyllids were introduced in the petri dish and psyllids began moving around, a single female adult *H*. *axyridis* was introduced into each petri dish. A similar sized pear leaf was placed in each petri dish arena and the petri dish was sealed with parafilm. Ten small holes were punched in the parafilm for ventilation with #1 insect pins. Predators were removed from the dishes after 24 hours, and the number of psyllids consumed in each petri dish was recorded. Each temperature treatment was replicated five times.

To compare the difference in predation capacity of *H*. *axyridis* on two morphotypes of psyllids, functional responses of *H*. *axyridis* to summerform and winterform adult psyllids were determined at 25°C. Predators usually show strong predatory efficiency to prey at 25°C [44,45]. The same experimental design was used as described above. Each morphotype treatment was replicated five times.

#### Weight and water content analysis of two morphotypes of *C*. *chinensis*

Winterform *C*. *chinensis* used to study water content and body weight were kept at 15 °C in laboratory conditions for 48h after collection from the field to keep the same biological status with those used in functional response experiments. Summerforms were kept under 25 °C laboratory conditions for 48h after collection from the field. The fresh weight of psyllids was measured with an electronic scale (TP-114, Denver Instrument, USA) with a group of ten live psyllids. Dry weight was obtained after drying these psyllids in an oven at 65°C until reaching a constant weight. Body water content of psyllids was calculated using fresh and dry weight. These samples were maintained at -80°C for the biochemical analyses described below. There were five replicates of each treatment.

#### Biochemical analysis of two morphotypes of *C*. *chinensis*

In order to study the mechanism of predation efficiency of *H*. *axyridis* between the two morphotypes, we measured the nutritional traits of both morphotypes. The total lipid, glycogen, trehalose and soluble protein of two morphotypes of psyllids were extracted and determined following the methods of Zhou *et al*. 2004 [46] and Shi *et al*. 2010 [47]. Total lipid content was examined using a vanillin assay. The anthrone method was used to measure the glycogen and trehalose content, and standards were purchased from Sigma Chemical Co. Soluble protein was determined followed the protocol of the BCA Protein Assay Kit (Biosynthesis Biotechnology Company, Beijing, China). The total lipid, soluble protein, glycogen, and trehalose content of adult psyllids were quantified separately by measuring optical densities at wavelengths of 525 nm, 562 nm, 625 nm, and 625 nm, respectively.

#### Cuticle analyses of two morphotypes of *C*. *chinensis*

During the experiment examining the functional response of *H*. *axyridis* on two morphotypes of psyllids, large numbers of prey corpses were found to be partially consumed. Generally, the prey abdomens were completely or partially consumed by *H*. *axyridis* while the head capsules and wings were left alone. Therefore, we assumed that the abdomen is the body part of *C*. *chinensis* that tends to be the most vulnerable and most easily attacked by *H*. *axyridis* Previous studies have demonstrated that cuticle thickness is a good measure of invertebrate intractability (structural strength, stiffness, or toughness of the prey to predators) [48,49]. Therefore, we tested thicknesses of the abdominal cuticles of psyllids to analyze the differences in intractability of the two morphotypes. Cuticle thickness measurement was performed according to the definition described in previous studies: that arthropod cuticle is composed of two layers, the outer protein-rich epicuticle and the procuticle (including exocuticle and endocuticle), which presents as a lamellar base structure based upon electron micrographs [50,51].

Samples of winterform and summerform psyllids used in transmission electron microscopy (TEM) were prefixed in a mixture of 2.5% glutaraldehyde. Samples were rinsed in PBS buffer (0.1M) three times for 15 min per rinse, then post-fixed in 1% osmium tetroxide for 1.5 h and rinsed three times again in PBS buffer. After fixation, the material was dehydrated in a series of ethanol with varying concentration (30%, 50%, 70%, 80%, 90%, 95%, 100%) and gradually embedded in epoxy resin after the pre-embedding stages [52]. Ultra-thin sections (approximately 70 nm thickness) of embedded material were cut on Leica Ultracut ultramicrotome (Wetzlar, Germany) with glass knives. Sections were mounted on 100 mesh copper grids and stained with aqueous uranyl acetate and lead citrate (standard recipe). TEM analyses were carried out using a Jeol JEM-1400 transmission electron microscope operated at 80 kV. The cuticle thicknesses of the first abdominal segment of the two morphotypes of *C*. *chinensis* were compared by using the program ImageJ (https://imagej.nih.gov/ij/) [53]. Five psyllids were measured for each morphotype.

#### Chitin assay of two morphotypes of *C*. *chinensis*

Chitin content of the psyllid adults was assayed by quantifying glucosamine (Sigma-Aldrich #G4875) derivatives obtained after deacetylation, depolymerization, and deamination of the N-acetyl-glucosamine polymer following the methods of Lehman and White 1975 and Farnesi *et al*. 2015 [54,55]. Before chitin quantification, weight of psyllids was determined to normalize the results. Briefly, 40 adult psyllids were homogenized in 0.5 mL of distilled water using an electric homogenizer. 0.5mL of distilled water for rinsing the grinding rod was combined with the homogenate and then centrifuged at 1800*g* for 15min at room temperature. To convert the chitin of the samples to chitosan, an alkaline digestion was performed by incubating the samples at 130°C for 1 h after the addition of 14M KOH. Later on, after reacting with HNO_2_, and with the addition of MBTH (Sigma-Aldrich #129739, 3-methyl-2-benzothiazolone hydrazone hydrochloride hydrate) and FeCl3·6H2O, soluble aldehydes were generated from depolymerized chitosan and deaminated glucosamine. 100 mL of each sample was transferred to a well in a 96-well microplate and then optical density (650 nm) was determined. Finally, chitin content was expressed as a glucosamine equivalent according to a standard curve obtained from various concentrations of glucosamine. Five replicates were measured for each morphotype.

### Statistical analysis

Functional response data were analyzed in two steps [56]. First, the type of functional response of *H*. *axyridis* on psyllids was examined by a logistic regression analysis of proportion of prey consumption (*Na/N*_0_) as a function of prey density (*N*_0_) using Eq (1) [56–59].

$$\frac{Na}{N_{0}}=\frac{\text{exp}(P_{0}+P_{1}N_{0}+P_{2}N_{0}{}^{2}+P_{3}N_{0}{}^{3})}{1+\text{exp}(P_{0}+P_{1}N_{0}+P_{2}N_{0}{}^{2}+P_{3}N_{0}{}^{3})}$$ (1)

where *Na* is the number of psyllids consumed; *N*_0_ is the initial psyllid’s density; and *P*_0_, *P*_1_, *P*_2_, and *P*_3_ are the intercept, linear, quadratic, and cubic coefficients, respectively. The type of functional response was determined by the negative or positive sign of the linear coefficient (*P*_1_) and the quadratic coefficient (*P*_2_) from the regression. A type I response has a linear coefficient where *P*_1_ = 0, a type II response has a *P*_1_ < 0, and a type III response has a *P*_1_ > 0 and the quadratic coefficient (*P*_2_) < 0 [60–64]. If estimates of the linear coefficient in the original model were not significantly different from 0, the model was reduced by omitting the cubic term until the coefficients were significant [56,65–66]. Logistic regression analysis using the glm function in program R [57] was used to estimate the values of *P*_0_, *P*_1_, *P*_2_, and *P*_3_.

The second step of the analysis was to use the Holling’s disc equation (Eq 2 shown below because all our results showed type II response) to estimate the parameters of the relationship between the numbers of psyllids consumed (*N*_a_) and psyllid density (*N*_0_). Handling time (*T*_*h*_) and attack rate (*a*) are usually used to estimate differences in functional response [63].

$$N_{a}=\frac{aTN_{0}}{1+aT_{h}N_{0}}$$ (2)

The parameters *a* and *T*_h_ were estimated by using non-linear least squares regression with the nls function in Program R [57,60,63,67]. The maximum number of attacked prey (*T/T*_*h*_) predicted by the functional response was also calculated. Shapiro-Wilk test was used to assess the assumption of normality, and Levene’s test was used to assessthe assumption of homogeneity of variances between the two morphotypes of *C*. *chinensis*. All assumptions were met, so we used a two-sample *t*-test in Program R to compare the differences in water content, body weight, lipid, glycogen, trehalose, protein, cuticle thickness, and chitin content between the two morphotypes. Generalized linear regression was used to study whether predation rates of predators were affected by temperature and psyllid morphotype.

## Results

### Predation capacity of *H*. *axyridis* on *C*. *chinensis*

**Fig 1:**
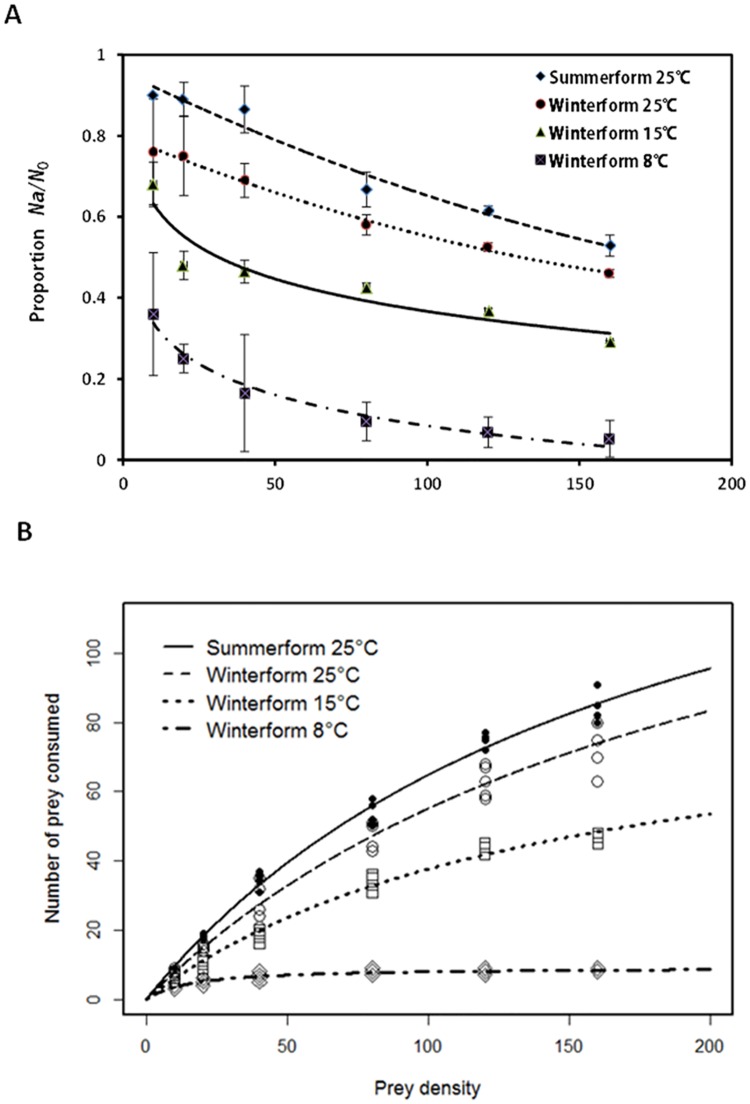
Proportions (A) and numbers (B) of the two morphotypes of *Cacopsylla chinensis* consumed by *Harmonia axyridis* adults with increasing prey density under different temperatures.

A negative *P*_*1*_ value (Table 1) indicated that the predation of summerform *C*. *chinensis* at 25°C and winterform *C*. *chinensis* at 8°C by *H*. *axyridis* conformed to a type II functional response. Because estimates of the linear coefficient in the original cubic model of the predation on winterforms under 15°C and 25°C were not significantly different from 0 (*p* > 0.05), we ran the reduced model. Similarly, a negative *P*_*1*_ value in the reduced model at 15°C (*P*_*1*_ = -0.07, *p*< 0.001) and 25°C (*P*_*1*_ = -0.0143, *p* = 0.002) indicated a type II functional response was a suitable fit for the predation of winterforms. The proportion of psyllids consumed by *H*. *axyridis* decreased monotonically with increased prey density under all temperatures (Fig 1A), which is the typical pattern of a type II functional response. The consumption rate of both summerform and winterform *C*. *chinensis* showed a decelerating rate with increasing prey density (Fig 1B) under all temperatures tested. We noted that in the lowest prey density treatments, *H*. *axyridi*s tended to consume all body parts of psyllids, but with increasing prey densities, more wings, heads, and thoraxes of psyllids were gradually left in the arena, indicating the saturating tendency of *H*. *axyridi*s.

**Table 1:** Logistic regression describing the proportion of two morphotypes of *Cacopsylla chinensis* consumed by *Harmonia axyridis* adults as a function of prey density on pear leaves.

Prey morphotype	Temp(°C)	Parameters	Estimate	SE	Z-value	Pr (>|z|)
**Summerform**	**25**	**P**_**0**_	**3.0920**	0.4998	6.188	0.000
		**P**_**1**_	**-0.0503**	0.0197	-2.543	0.011
		**P**_**2**_	**0.0003**	0.0002	1.507	0.132
		**P**_**3**_	**-0.0000009**	0.0000008	-1.162	0.245
**Winterform**	**25**	**P**_**0**_	**1.4720**	0.3544	4.153	0.000
		**P**_**1**_	**-0.0227**	0.0154	-1.468	0.142
		**P**_**2**_	**0.0001**	0.0002	0.739	0.460
		**P**_**3**_	**-0.0000004**	0.0000007	-0.51	0.568
**Winterform**	**15**	**P**_**0**_	**0.5631**	0.3157	1.783	0.0755
		**P**_**1**_	**-0.0247**	0.0144	-1.716	0.0862
		**P**_**2**_	**0.0002**	0.0002	1.302	0.1929
		**P**_**3**_	**0.0000008**	0.0000006	-1.326	0.184
**Winterform**	**8**	**P**_**0**_	**-0.2270**	0.3800	-0.597	0.5516
		**P**_**1**_	**-0.0466**	0.0197	-2.364	0.0181
		**P**_**2**_	**0.0003**	0.0003	1.296	0.1958
		**P**_**3**_	**0.0000009**	0.0000010	-0.969	0.333

Based on the Holling II equation, we calculated handling time, attack rate, and the theoretical maximum number of two psyllid morphotypes consumed by *H*. *axyridis* under different temperature settings (Table 2). *H*. *axyridis* consumed greater proportion of summerforms at 25°C compared with winterforms (Fig 1A). *H*. *axyridis* showed a higher attack rate, higher estimated maximum prey, and shorter handling time on summerforms than winterforms (Table 2). Therefore, *H*. *axyridis* presented stronger predation capacity on summerform *C*. *chinensis* than winterforms at 25°C. The predation capacity of *H*. *axyridis* was higher under warmer temperatures compared with the cooler temperatures. Likewise, a higher attack rate (0.64±0.04) and shorter handling time (0.01±0.00d) were observed at 15°C compared with 8°C for winterforms (Table 2). *H*. *axyridis* adults preyed on winterform *C*. *chinensis* at both temperatures representing early-spring conditions (8°C and 15°C). The estimated maximum number of prey that could be consumed by *H*. *axyridis* at 15°C was up to 92.59 (Table 2), suggesting the great potential of using *H*. *axyridis* to control winterform *C*. *chinensis* in early-spring. The greatest predation capacity of winterform psyllids by *H*. *axyridis* was found at 25°C (Table 2, Fig 1B). We noted that temperature was impactful on predation capacity of *H*. *axyridis*, perhaps more so than morphotype. Therefore, we analyzed the predation data using generalized regression analysis. The results of generalized regression showed that when accounting for temperature (p< 0.0001), prey morphotype still significantly influenced the number of psyllids consumed by *H*. *axyridi*s (p = 0.0003), indicating that both morphotype and temperature mattered.

**Table 2:** Mean (±SE) attack rates (*a*), handling times (*T*_*h*_), and theoretical maximum number (*N*_*max*_) of *Harmonia axyridis* adults preying on two morphotypes of *Cacopsylla chinensis*.

Prey morphotype	Temp(°C)	*a*	*T*_*h*_ (d)	*N*_*max*_	R^2^
**Summerform**	**25**	1.0056±0.0392	0.0055±0.0004	181.82	0.99
**Winterform**	**25**	0.8141±0.0579	0.0059±0.0007	169.49	0.97
**Winterform**	**15**	0.6357±0.0359	0.0108±0.0008	92.59	0.98
**Winterform**	**8**	0.5668±0.0660	0.1082±0.0038	9.24	0.85

### Body weight and water content of two morphotypes

**Fig 2:**
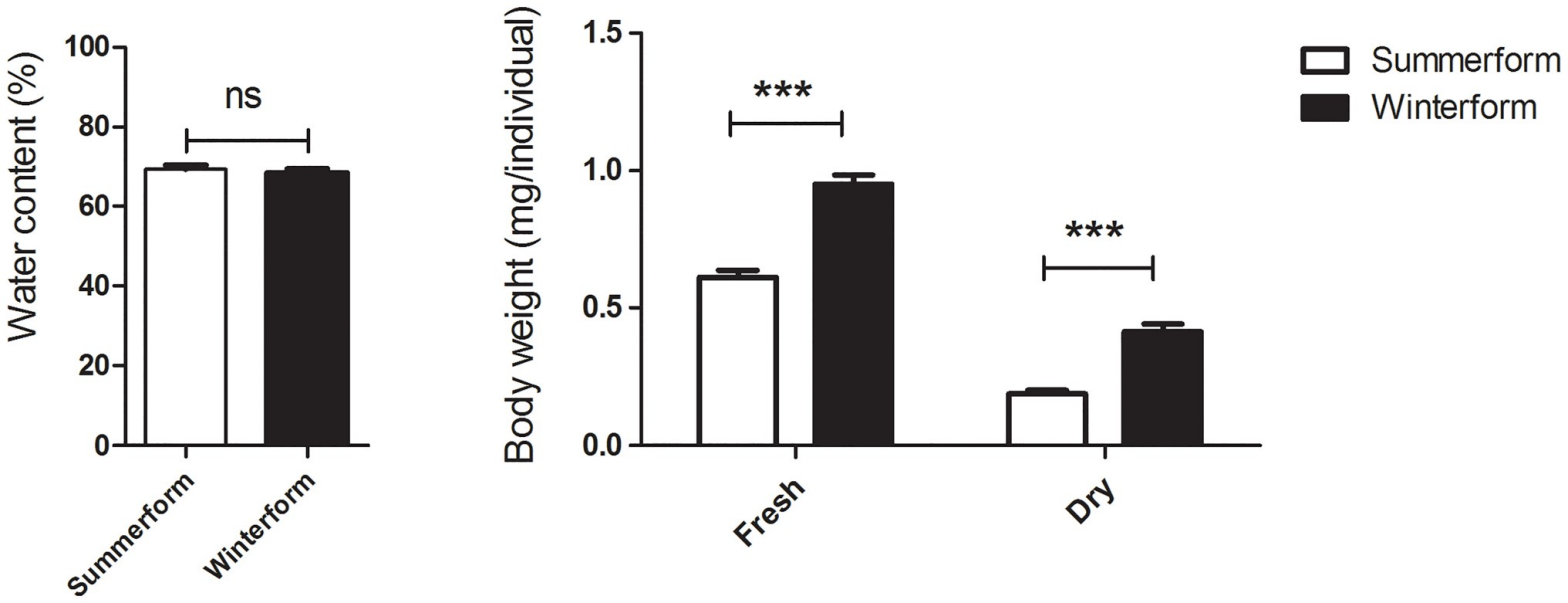
Water content, fresh, and dry body weights of two morphotypes of *Cacopsylla chinensis*. Bars denote SE; *** (*p*<0.001, two sample *t*-test).

Water content did not differ between summerform (69.36% ±2.23%, two-sample t-test, *t*_8_ = 0.591, *p*>0.05; Fig 2) and winterform psyllids. Winterform psyllids had significantly higher fresh weight (1.31±0.13 mg, two-sample *t*-test, *t*_8_ = 7.89, *p*< 0.001; Fig 2) and dry weight (0.41 ±0.06mg, two-sample *t*-test, *t*_8_ = 7.45, *p*< 0.001; Fig 2) than summerforms. The dry weight of winterform psyllids was127.8% higher than the weight of summerform psyllids, possibly due to the larger body size of winterforms.

### Biochemical analysis of two morphotypes

**Fig 3:**
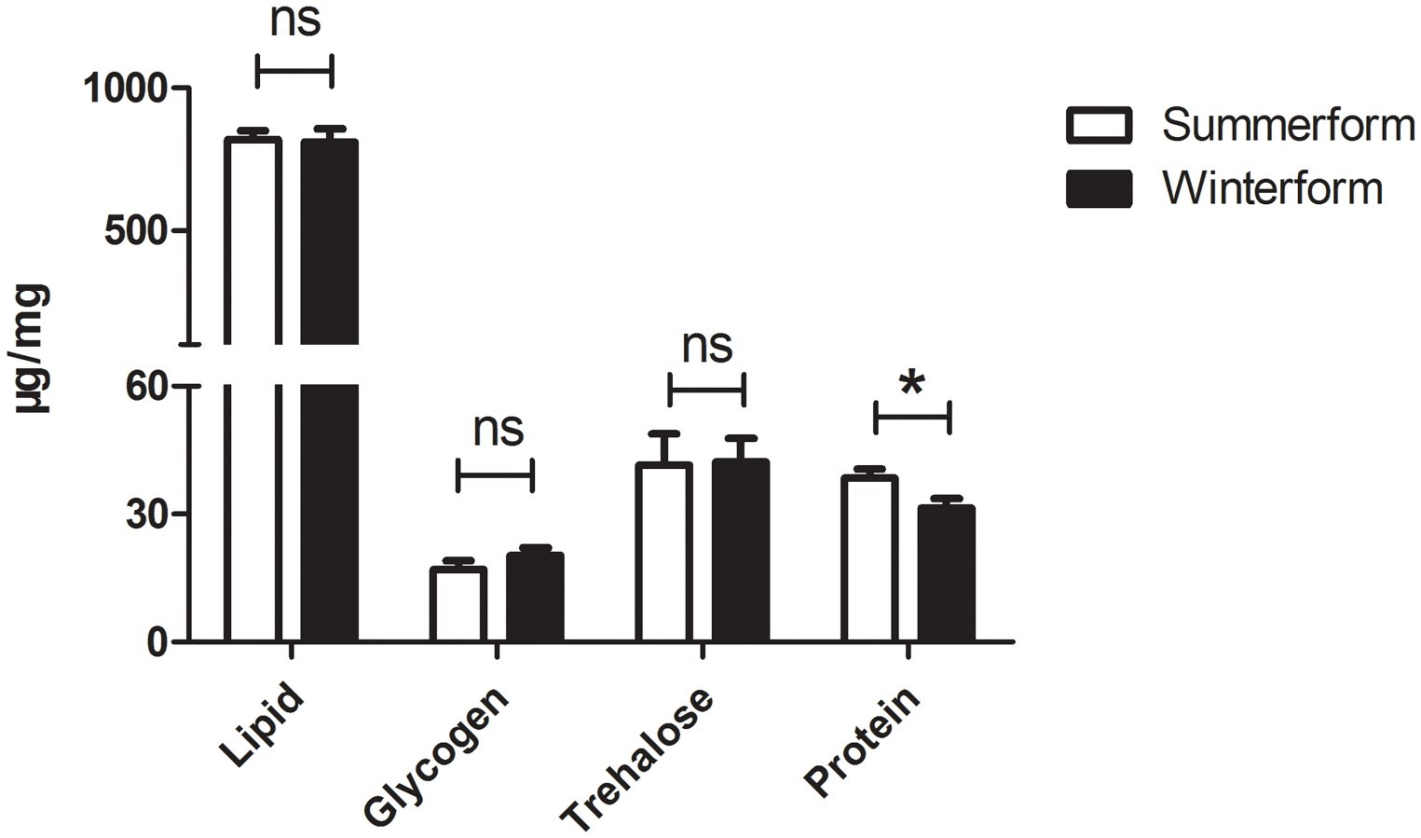
Total lipid, glycogen, trehalose and soluble protein content (μg/mg dry weight^-1^) of summerform and winterform of *Cacopsylla chinensis*. Bars denote SE; * (*p* < 0.05, two sample *t*-test).

Because of the bodyweight difference of the two morphotypes, we normalized each measurement by body weight. Soluble protein level in summerform *C*. *chinensis* (38.69±4.67μg/mg, two-sample *t*-test, t_8_ = 2.400, *p* = 0.043) was higher than winterforms (Fig 3). Although not statistically significant, summerform psyllids exhibit a slightly higher level of total lipid (two-sample *t*-test, t_8_ = 0.181, *p* = 0.861), but lower glycogen (two-sample *t*-test, t_8_ = -1.16, *p* = 0.280) and trehalose (two-sample *t*-test, t_8_ = -0.075, *p* = 0.942) levels than winterforms (Fig 3).

### Cuticle analyses of two morphotypes

**Fig 4:**
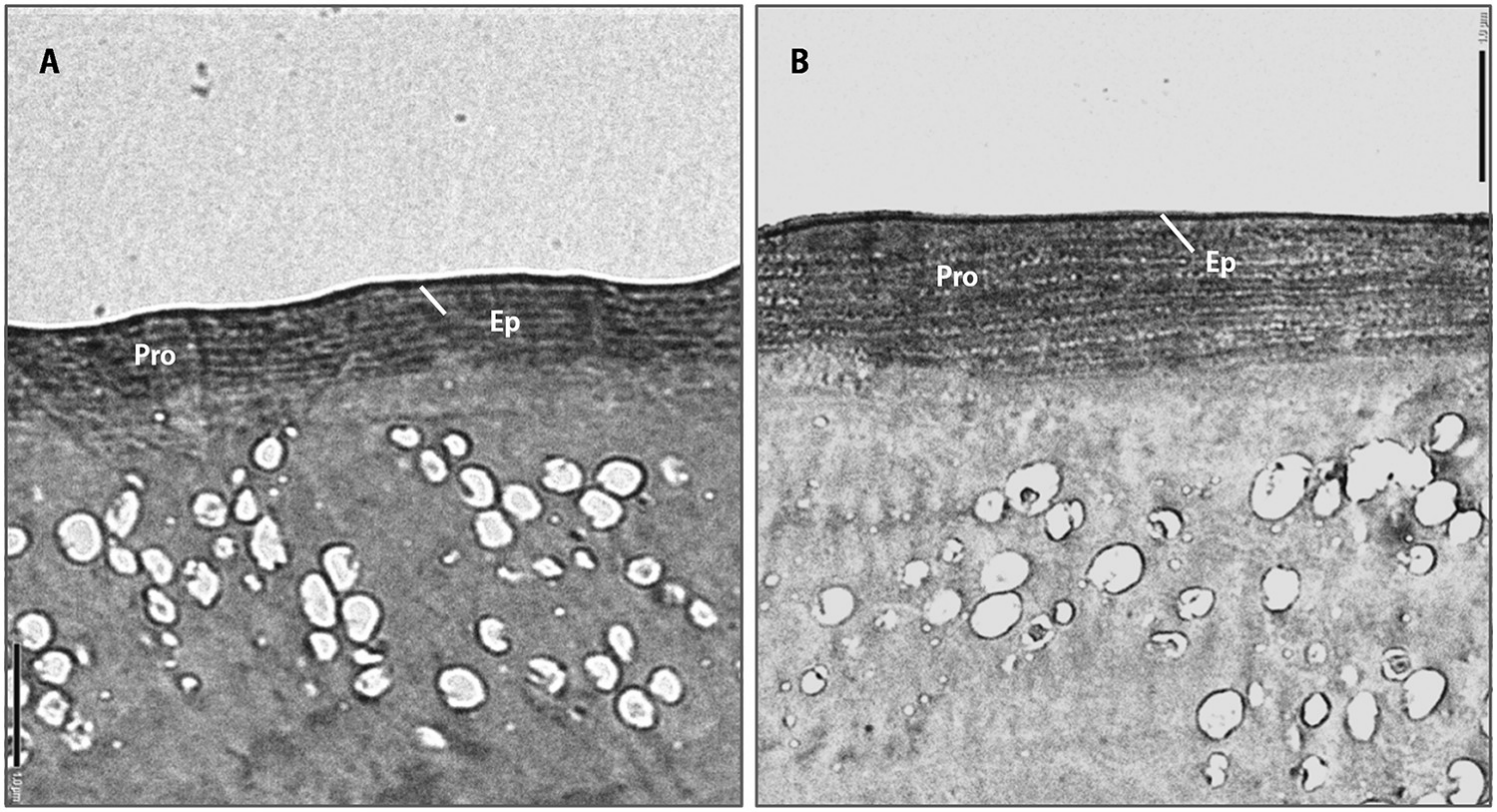
Ultrastructure of the abdominal cuticle of summerform and winterform of *Cacopsylla chinensis*. A: summerform of *C*. *chinensis*; B: winterform of *C*. *chinensis*. Ep: epicuticle; Pro: procuticle; measuring scales shown in both photos are 1.0μm.

**Fig 5:**
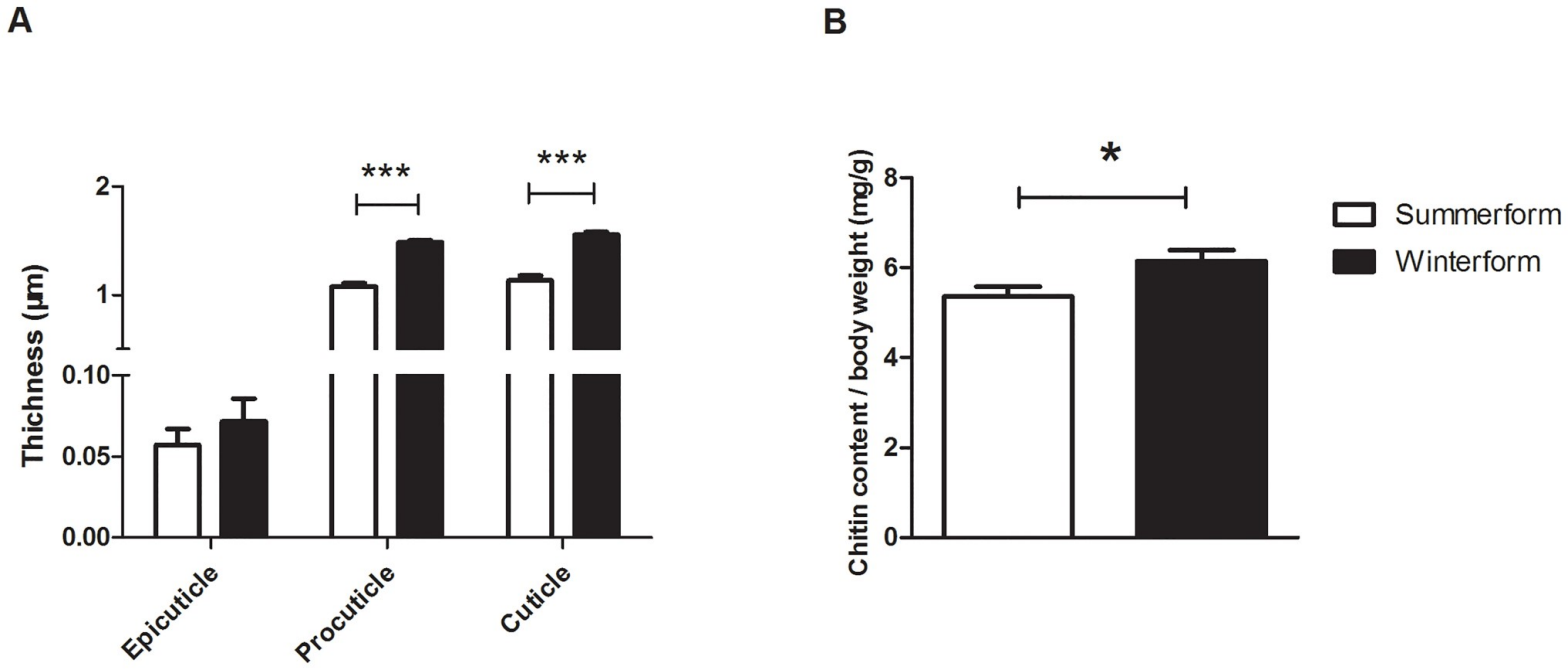
Cuticle thickness and chitin content (mg/g body weight^-1^) of summerform and winterform of *Cacopsylla chinensis*. A: cuticle thickness; B: chitin content (mg/g body weight^-1^). Bars denote SE; * (*p* < 0.05, two sample *t*-test), ** (*p* < 0.01, two sample *t*-test), *** (*p* < 0.001, two sample *t*-test).

The overall cuticle thicknesses of the first abdominal segment of winterforms were higher than summerforms (Figs 4 and 5A). We found the same trend between two morphotypes that epicuticle thickness (0.07±0.01μm, two-sample *t*-test, t_8_ = 0.865, *p* = 0.412; Fig 5A), procuticle thickness (1.48±0.02μm, two-sample *t*-test, t_8_ = 10.825, *p*< 0.001; Fig 5A) and total cuticle thickness (1.56±0.02μm, two-sample *t*-test, t_8_ = 8.810, *p*< 0.001; Fig 5A) was higher on winterform psyllids.

### Chitin assay of *C*. *chinensis*

We found that winterform psyllids (6.15±0.50mg/g, two-sample *t*-test, *t*_7_ = 2.41, *p* = 0.047; Fig 5B) exhibited higher chitin level compared to that of summerforms. This is positively related to the cuticle thickness on winterforms. Thus, the higher chitin level in winterforms was likely due to the chitin-rich procuticle, which accounted for the primary proportion of total cuticle. Results of cuticle thickness and chitin level indicated winterform psyllids have harder and thicker exoskeletons than summerform psyllids.

## Discussion

In this study, we examined the functional response of *H*. *axyridis*, a ubiquitous predator of the two morphotypes of *C*. *chinensis*. Predation of *H*. *axyridis* on winterform *C*. *chinensis* was positively related to increases in temperature from 8°C to 25°C, as is also the case in other predaceous insects [21]. Our results have shown that both the morphotype of *C*. *chinensis* and temperature impact the functional response of *H*. *axyridis*. However, our experiments on variation in predation capacity under different temperature conditions were limited to. Further experiments on the effect of temperature on predation of summerform *C*. *chinensis* are still required.

A higher rate of predation was found for summerform than winterform psyllids (Fig 1B). The difference in predation capabilities was highly due to the morphological and biochemical variations between two morphotypes. First, our results indicated that winterforms weighed more than summerforms, the body weight difference can be explained by larger body sizes, which is often the case for pear psyllids [26–27,68]. The size of prey insects is an important factor influencing the foraging behavior of their natural enemies, including encounter rate and prey-handling ability [69]. Thus, the larger body size of winterforms may account for lower predation by *H*. *axyridis*. Second, in our study, summerform *C*. *chinensis* had higher levels of protein content than the winterforms. Nutritional level of prey has long been recognized to affect predatory behavior [32,33,70]. Relative amounts of nutrients in diet affect the activities of carnivores as well as their performance on survival, foraging, development, reproduction and distribution [33–35]. Insect predators can regulate their intake of various nutrients to fit their needs. Proteins and lipids are the two most important and well-studied components of predators’ diets [24–26]. Preys containing more nitrogen and protein are usually considered a higher quality diet for various generalist predators. Previous studies have found that predators select prey to optimize their essential amino acids intake and that predators are adapted to protein-rich diets [32–33]. The prey consumptions of most predators have been found to exceed their energetic demands [32]. Feeding habits of predators are also related to their genetically inheritable preference [71]. Summerform psyllids may better meet *H*. *axyridis* nutritional requirements and intrinsic demands compared to winterforms, leading to greater consumption of summerforms. Third, we found thinner exocuticles and lower chitin levels in summerforms than winterforms. The chitin-rich procuticle forms the basis of the exoskeleton and provides mechanical support and rigidity to protect to the insect body [72–73]. This makes chitin level a good indicator of the “hardness” of insect exoskeletons where greater chitin levels increase exoskeleton “hardness”. [73]. Analyses of cuticle thickness and chitin content indicated that summerform psyllids have softer and thinner exoskeleton than winterforms. Predators often prefer prey which are easier to digest like those with softer bodies because of the lower energy cost invested during foraging and handling [48,74]. The relatively lower energy consumption of predators when dealing with summerforms might be the reason that *H*. *axyridis* exerted higher predatory efficiency. It has been documented that invertebrate and insectivorous vertebrate predator species alter foraging behaviors due to cuticle thickness or “hardness” of the prey [36,37]. Optimal foraging theory predicts that for natural enemies, decision of whether to attack an encountered prey or not is based on the potential gain from searching for a new prey item [75]. Predators often select prey with the best energy return [13,74].

Previous studies have shown that differences between the two morphotypes of pear psylla were regulated by environmental factors [27,76–79], e.g. pear psylla *C*. *bidens* (Šulc) and *C*. *pyricola* (Förster) became larger and darker under laboratory manipulation with shorter photoperiod and lower temperature [27]. Glycogen is the main metabolic fuel and source for trehalose and polyol synthesis, glycogen and trehalose were usually thought to help overwintering psyllids survive in winter [29,78]. It was found that in pistachio psylla, *Agonoscena pistaciae* (Burckhardt & Lauterer), the changes in energy allocation related to ambient temperatures [29]. We may not have found differences in glycogen and trehalose between the two morphotypes because the winterform psyllids were collected in March, while the most extreme energy allocation in overwintering *C*. *chinensis* may occur in December and January. The morphological and physiological variation between seasonal morphotypes is likely the product of the adaptation to different environmental conditions and predation pressures. Insect prey evolve various defense mechanisms against their predators [79]. Some of the defensive traits could change over time [80]. It has been documented that when predators were present, prey developed tougher and heavier exoskeletons as morphological defenses, e.g. mayflies and dragonflies [81–82]. The thicker exoskeleton of winterform *C*. *chinensis* might be attributed to a plastic response to the presence of predators. Thus, we hypothesize that winterform psyllids are less desirable prey than summerform psyllids because of their greater energy allocation to defense against predators. Further, features that increase overwintering survival may render winterform psyllids less nutritious to their predators. However, this hypothesis remains to be tested in future studies.

Data on the functional responses of predators, while useful, can’t fully predict foraging behaviors of predators in the field [41,83]. Spatial complexity in field might account for the discrepancy of functional responses under field and laboratory conditions [84]. Other studies have also demonstrated that the functional responses in laboratory could partly reflect the real scenario in the field especially under high prey infestation [61]. However, our results provided valuable evidence supporting the potential of incorporating *H*. *axyridis* as an effective biocontrol agent in management programs of pear orchards. We noted that more partially consumed corpses of both morphotypes were found in our experiments with increasing prey density, suggesting that *H*. *axyridis* killed more pear psylla than they can consume and have a tendency to reach saturation. Similar findings were reported for cotton aphid *Macrosiphum euphorbiae* (Thomas), fruit flies *Drosophila melanogaster*, and house crickets *Acheta domestica* (L.) [85–87]. The ‘superfluous killing’ indicates the adaptation of predators to food-limited environments [87]. We did not observe any behavioral or morphological changes of *H*. *axyridis* after feeding on the two morphotypes of pear psyllids which might due to the high prey suitability of *H*. *axyridis* [88]. Our future work would investigate the long-term effect of the two psyllid morphotypes on the development and fecundity of *H*. *axyridis*. Predators demonstrating type II functional response can be incorporated in inundative biological control of direct pest suppression for short term [89]. Because of the large influence of temperature on the predation rate of winterform *C*. *chinensis* by *H*. *axyridis* and the Type II functional response of *H*. *axyridis* to pear psylla, temperature during inundative release of *H*. *axyridis* against *C*. *chinensis* in the field should be taken into account. Control of winterforms could maximize the psyllid suppression by reducing annual damage on new leaves and branches. In addition, based on the great control potential of *H*. *axyridis* on *C*. *chinensis* and its ubiquity in fruit orchards globally, the effect of *H*. *axyridis* on other species of pear psylla is worth further investigation. Broad spectrum chemical pesticides should be avoided in pear orchards even after fruit harvest to facilitate the development of *H*. *axyridis* population [7,11]. These strategies will benefit sustainable pear production and reduce safety concerns from the social-environmental perspective.

## Conclusion

Predation capabilities of *H*. *axyridis* on winterform *C*. *chinensis* were studied under different early-season temperatures and results indicated high consumption of *C*. *chinensis* at 8°C and 15°C. Higher predation capacity of summerform *C*. *chinensis* was found than that of winterforms, shown as higher attack rate, shorter handling time as well as higher theoretical maximum number consumed by *H*. *axyridis*. We found that the stronger predation of *H*. *axyridis* on summerforms was likely due to the higher nutritional returns, e.g. higher protein content and less foraging costs when dealing with summerforms, smaller size, and softer and thinner exoskeleton. The high predation capacities of *H*. *axyridis* on both morphotypes of psyllids indicated its great potential as a biocontrol agent of *C*. *chinensis*. Based on the inverse density-dependent predation of type II functional response and the great predation capacities of *H*. *axyridis* on the two morphotypes, the control of *C*. *chinensis* should start early in the season to suppress the winterforms by inundative release. Further study of the control efficiency of two morphotypes of *C*. *chinensis* by *H*. *axyridis* in the field is necessary to extend our findings to on-farm pear psylla management.

## Supporting information

S1 Fig Relation between the biochemical parameters and the predation capacity of *H*. *axyridis*. (TIF)
