# Supplementary figures and images for: Different predation capacities and mechanisms of Harmonia axyridis (Coleoptera: Coccinellidae) on two morphotypes of pear psylla Cacopsylla chinensis (Hemiptera: Psyllidae)
Source: PLoS One. 2019 Apr 23;14(4):e0215834. doi: 10.1371/journal.pone.0215834 (PMC6478344; doi:10.1371/journal.pone.0215834)

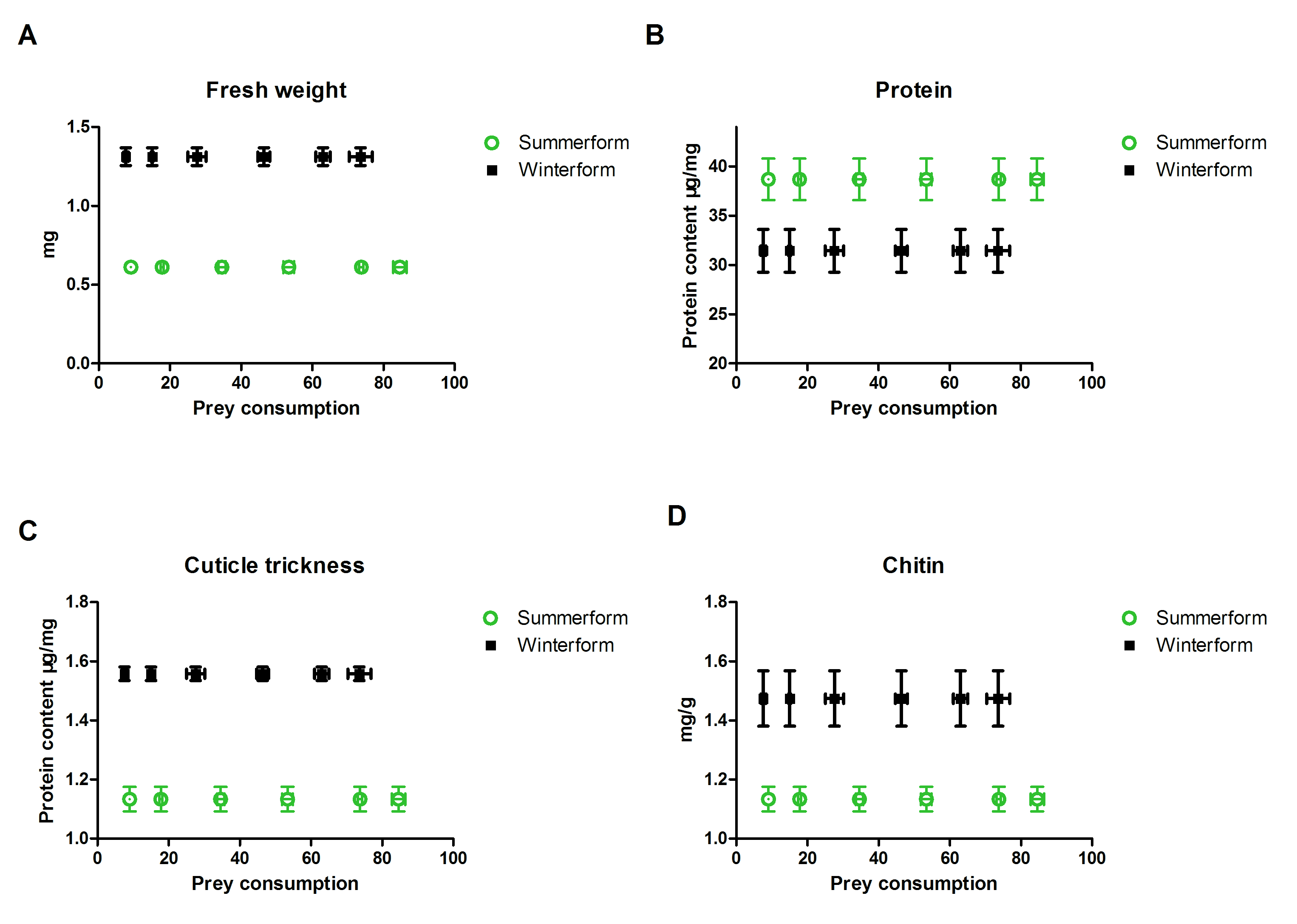

Supplement: S1 Fig — (TIF) [file pone.0215834.s001.tif]
